# Classification of negative and positive ^18^F-florbetapir brain PET studies in subjective cognitive decline patients using a convolutional neural network

**DOI:** 10.1007/s00259-020-05006-3

**Published:** 2020-09-02

**Authors:** Bart Marius de Vries, Sandeep S. V. Golla, Jarith Ebenau, Sander C. J. Verfaillie, Tessa Timmers, Fiona Heeman, Matthijs C. F. Cysouw, Bart N. M. van Berckel, Wiesje M. van der Flier, Maqsood Yaqub, Ronald Boellaard, Alzheimer’s Disease Neuroimaging Initiative

**Affiliations:** 1 Department of Radiology and Nuclear Medicine, Amsterdam UMC, Vrije Universiteit Amsterdam, De Boelelaan, 1117 1081 HV Amsterdam, The Netherlands; 2 Alzheimer Center and department of Neurology, Amsterdam UMC, Vrije Universiteit Amsterdam, De Boelelaan, 1117 1081 HV Amsterdam, The Netherlands; 3 Department of Epidemiology & Biostatistics, Amsterdam UMC, Vrije Universiteit Amsterdam, De Boelelaan, 1117 1081 HV Amsterdam, The Netherlands

**Keywords:** Convolution neural network, Artificial intelligence, Subjective cognitive decline, Classification, Amyloid, ^18^F-florbetapir

## Abstract

**Purpose:**

Visual reading of ^18^F-florbetapir positron emission tomography (PET) scans is used in the diagnostic process of patients with cognitive disorders for assessment of amyloid-ß (Aß) depositions. However, this can be time-consuming, and difficult in case of borderline amyloid pathology. Computer-aided pattern recognition can be helpful in this process but needs to be validated. The aim of this work was to develop, train, validate and test a convolutional neural network (CNN) for discriminating between Aß negative and positive ^18^F-florbetapir PET scans in patients with subjective cognitive decline (SCD).

**Methods:**

^18^F-florbetapir PET images were acquired and visually assessed. The SCD cohort consisted of 133 patients from the SCIENCe cohort and 22 patients from the ADNI database. From the SCIENCe cohort, standardized uptake value ratio (SUVR) images were computed. From the ADNI database, SUVR images were extracted. 2D CNNs (axial, coronal and sagittal) were built to capture features of the scans. The SCIENCe scans were randomly divided into training and validation set (5-fold cross-validation), and the ADNI scans were used as test set. Performance was evaluated based on average accuracy, sensitivity and specificity from the cross-validation. Next, the best performing CNN was evaluated on the test set.

**Results:**

The sagittal 2D-CNN classified the SCIENCe scans with the highest average accuracy of 99% ± 2 (SD), sensitivity of 97% ± 7 and specificity of 100%. The ADNI scans were classified with a 95% accuracy, 100% sensitivity and 92.3% specificity.

**Conclusion:**

The 2D-CNN algorithm can classify Aß negative and positive ^18^F-florbetapir PET scans with high performance in SCD patients.

## Introduction

Patients with subjective cognitive decline (SCD) are at increased risk for developing mild cognitive impairment (MCI), Alzheimer’s disease (AD) or other types of dementia [1, 2]. In the diagnostic process of patients with SCD, amyloid imaging can be used in order to assess the presence and extent of amyloid-beta (Aß) depositions in vivo [3–6]. Currently, the gold standard for determining the presence of such depositions in a clinical setting is a dichotomous visual assessment performed by a trained reader. However, accurate classification depends on training and experience of the reader and visual assessment can be challenging, in particular, for low levels of Aß depositions, as may be the case in patients with SCD.

In the past decade, various computer-aided pattern recognition algorithms have been developed to evaluate and identify PET patterns associated with specific disease stages, based on ^18^F-fluoro-deoxyglucose (^18^F-FDG) brain PET images [7, 8]. These studies applied machine learning approaches using atlas-based anatomical volumes of interest (VOIs) for feature extraction to classify AD progression in PET images. Despite yielding good results, feature extraction depending on VOI parcellation can be time-consuming, prone to MRI segmentation errors and is observer dependent in case of manual delineations. Furthermore, disease-specific patterns might not follow predefined VOIs. Thus, deep learning algorithms, such as a convolutional neural network (CNN), may provide superior performance since no a priori VOI definition or segmentation is required. Furthermore, with the amount of computational power currently available, CNNs are able to address the increasing complexity and quantities of imaging data, while providing both deterministic and objective results. Recently, CNNs have been effectively applied in ^18^F-FDG-PET neurodegeneration studies to discriminate between diagnostic groups and identify the patterns related to AD progression without the use of pre-defined VOIs [9]. However, in case of patients with SCD, feature extraction can be more difficult than in AD patients, since Aß depositions can be subtle in relation to non-specific background uptake. Therefore, it is of interest whether CNN also could effectively be applied in ^18^F-florbetapir PET studies in patients specifically with SCD.

The aim of this study was to develop, train, validate and test a 2D-CNN which is able to classify Aß negative (i.e. no amyloid accumulation) and positive (i.e. with amyloid accumulation) ^18^F-florbetapir PET scans in patients with SCD.

## Methods and materials

### Participants

A total of 133 SCD subjects from the Subjective Cognitive ImpairmENt Cohort (SCIENCe) study [2] (for training and validation) were included in this study. The SCIENCe project is a longitudinal observational study, with yearly assessments, to investigate the earliest changes related to AD. Prior to inclusion, all SCIENCe subjects underwent standardized dementia screening according to the procedures of the Amsterdam Dementia Cohort [10]. Individuals were labelled as SCD when they experienced cognitive complaints but could not be diagnosed with MCI, dementia or any other disease which is known to cause memory complaints. Inclusion criteria for the SCIENCe cohort are a diagnosis of SCD and age ≥ 45 years [2]. Before enrolment, all SCIENCe subjects provided written informed consent and the studies were approved by the Medical Ethics Review Committee of Amsterdam UMC, location VUmc.

A total of 22 SCD subjects (used as fully independent external test data) used in the preparation of this article were obtained from the Alzheimer’s Disease Neuroimaging Initiative (ADNI) database (adni.loni.usc.edu). The ADNI was launched in 2003 as a public-private partnership, led by Principal Investigator Michael W. Weiner, MD. The primary goal of ADNI has been to test whether serial magnetic resonance imaging (MRI), positron emission tomography (PET), other biological markers, and clinical and neuropsychological assessment can be combined to measure the progression of mild cognitive impairment (MCI) and early Alzheimer’s disease (AD).

### Data acquisition

SCIENCe PET data were acquired on a Gemini or Ingenuity TF PET/CT scanner (Philips Medical Systems, Best, the Netherlands) and head movements were minimized by using a head holder. First, a low-dose computed tomography (CT) scan was acquired for attenuation and scatter corrections. Next, directly after tracer injection (370 MBq to 425 MBq), a 90-min dynamic ^18^F-florbetapir PET scan was obtained, consisting of 29 frames (1 × 15, 3 × 5, 3 × 10, 4 × 60, 2 × 150, 2 × 300, 4 × 600 and 10 × 300 s), and raw data were reconstructed using line-of-response row-action maximum likelihood algorithm (LOR-RAMLA) (Gemini) or ordered-subsets time of flight (BLOB-OS-TF) (Ingenuity). During reconstruction, corrections for decay, dead time, normalization (detector sensitivities), attenuation, random coincidences and scatter were applied. The reconstructed images had a matrix size of 128 × 128 × 90 and a voxel size of 2.0 mm in all 3 directions. T1-weighted MR images were acquired at 3.0 Tesla using either an Ingenuity TF PET/MR (Philips Medical Systems, Cleveland, Ohio, USA) or a Signa HDxt MRI (General Electric, Milwaukee, WI, USA) scanner for structural information.

In the ADNI database, PET image acquisition has been done according to standard ADNI acquisition protocol [11]. The scans had a matrix size of 160 × 160 × 96 and a voxel size of 1.5 mm in all 3 directions. The ADNI PET studies used for the external test cohort in this study were acquired from 15 different centres.

### Image processing

The T1-weighted MR images (acquired within the SCIENCe project) were co-registered to the (dynamic) PET scans using VINCI (Max Planck Institute for Metabolism Research, Köln, Germany) as described previously [12–14]. Next, PVElab [15] software was used, together with the Hammers template [16], to extract reference tissue (grey matter cerebellum) time-activity curves (TACs). The grey matter cerebellar TAC was then used in combination with the dynamic PET image to calculate SUVR images from 50 to 70 min p.i. [17, 18]. These SUVR images were then spatially aligned using Statistical Parametric Mapping (SPM8) [19] using the T1-weighted MR images and a standard brain T1-template atlas from the Montreal Neuroimaging Institute (MNI). Next, because of missing MRI data from the ADNI data, an average PET template was acquired by averaging ten Aß negative and positive (5/5) ^18^F-florbetapir spatially aligned PET scans of the SCIENCe cohort. The ADNI PET scans were then spatially aligned using SPM8 using this average PET template. The resulting images (SCIENCE and ADNI) had a matrix size of 79 × 95 × 68 and a voxel size of 2.0 mm in all three directions. Voxels outside the brain were avoided from analyses using whole brain grey and white matter templates (MNI).

### CNN data preparation and augmentation

All training and validation PET scans were visually assessed and labelled (positive or negative) by an experienced nuclear medicine physician (BB). The external ADNI test set was visually assessed, labelled and a confidence score [low to high:1–5] was given by two qualified ^18^F-florbetapir readers (BB and SV).

Because of the high frequency of amyloid negative cases, and imbalance of the two groups (positive or negative), oversampling of the minority class was performed in training, to avoid the model to be biased towards the majority class [20]. Furthermore, data augmentation was applied (using random rotation, shift, shear zooming and flipping) to artificially create new PET images and to make the neural network more robust against head orientation [21]. To reduce complexity and circumvent the computational and memory requirements necessary for CNN-based classification of 3D PET images, stacked 2D PET images (axial, coronal and sagittal slices) were used instead.

### Model architecture

**Fig. 1:**
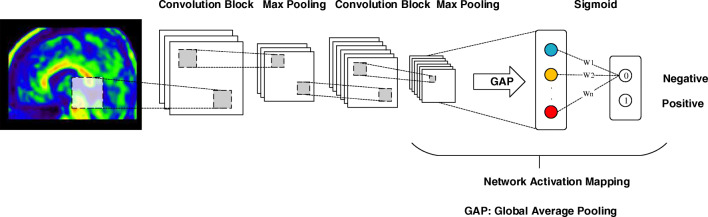
Architecture of the CNN. Each convolution block consists of two convolution layers, batch normalization and two ReLu activation functions. Max pooling is performed to down sample the data. Using a sigmoid function weights (Wn) are added to the nodes generated by the GAP layer. Network activation mapping is applied for object localization

CNNs are able to learn latent and generic features from the image slices [9, 22, 23]. This study proposes a 2D-CNN framework to extract intra-slice features from the 2D image slices. For each slice, here called decomposition (axial, coronal and sagittal), a 2D-CNN model is constructed. The 2D-CNN architecture consists of two convolution blocks consisting each of two convolution layers to extract image features [24], four Rectified Linear Unit (ReLu) activation layers to introduce non-linear properties to the model [25], two max-pooling layers to down-sample input representation [26], batch normalization to make the model converge faster [27], a global average pooling layer for object localization [28] and a final sigmoid dense layer to obtain a classification (Fig. 1). Dropouts are commonly used to avoid overfitting and were implemented after the fully connected layers. However, by implementing them after the max-pooling layers, artificial noise is created, to improve generalization of the trainable features. Therefore, two dropouts of 60% per epoch were implemented after the max-pooling layers [29].

The proposed models were implemented in the Keras library in Python (version 3.6), using TensorFlow as backend. For weights optimization, an Adam optimizer was used with a low learning rate of 1 × 10^−5^ with a decay of 1 × 10^−6^. Furthermore, the batch size for training the CNNs was set to the size of the full training dataset.

### Model performance

**Fig. 2:**
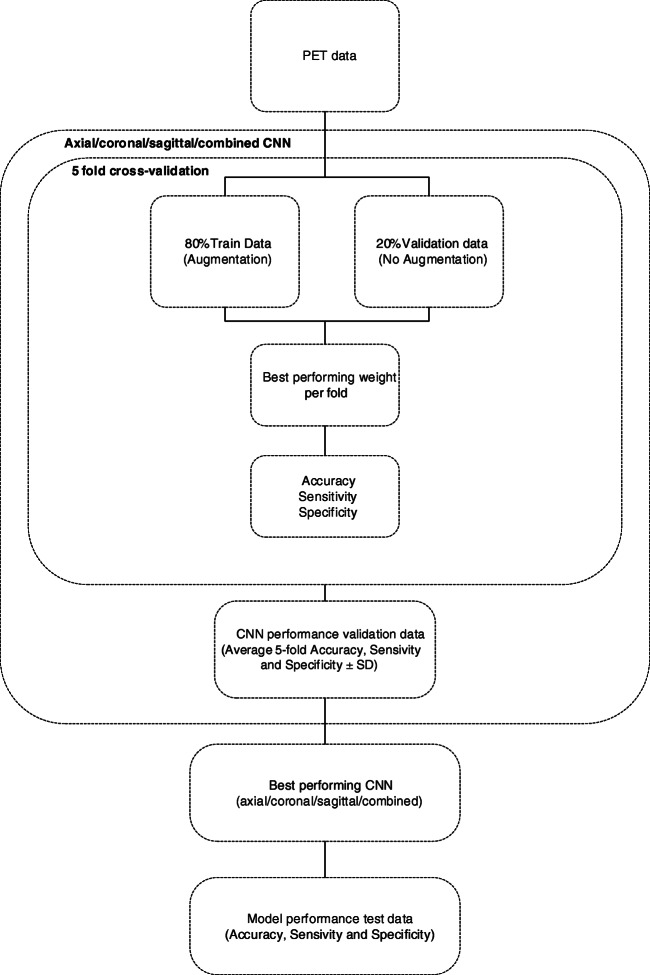
Schematic overview of the deep learning pipeline. The CNN uses a 5-fold cross-validation, where for each fold 80% of the data is used for training and 20% for validating the CNN. The best performing CNN is defined based on the average 5-fold accuracy, sensitivity and specificity and tested on an external dataset

**Fig. 3:**
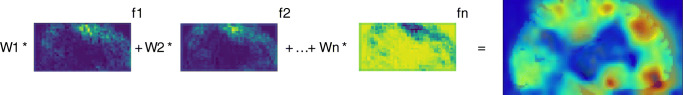
Network activation mapping. The global average pooling layer takes the average of each of the filters (fn) of the last max-pooling layer. The activation dense layer determines the individual weights (Wn) of each of these global average pooling nodes, resulting in a class prediction

A stratified five-fold cross-validation was used to evaluate the performance of each model its accuracy, sensitivity and specificity (Fig. 2) [30]. To this end, the SCIENCe dataset was split into five groups, instantiating five weights/models for each CNN (15 in total); four groups were each fold used for training purposes and the fifth for validation of the model. Next, the validation accuracy, sensitivity and specificity were averaged to obtain a reliable performance measure per CNN. The model that performed highest using the SCIENCe dataset was considered the best model. In addition, the three individual CNN predictions (axial, coronal and sagittal) were majority-hard-voted to obtain a combined classification. Last, the external ADNI dataset was used to assess the performance of the best model in an independent dataset. To determine whether similar spatial patterns are important for the CNN as for the readers, network attention area maps were obtained (see Fig. 3) [28]. The spatial patterns were detected by the global average pooling layer, which averaged contributions of each of the patterns in the feature maps from the convolution layers. More specifically, the nodes considered most important for classification received a higher weight (Wn) from the activation sigmoid dense layer, as visualized in the network attention area map.

## Results

In Table 1, demographic and clinical data is presented. Table 2 shows the performance of the different CNNs on classification of Aβ positive and negative ^18^F-florbetapir scans for both the SCIENCe (training and validation) and ADNI (test) dataset. No overfitting occurred during training; given that differences in model performance between training and validation dataset were small. In general, best results were seen with a 2D-CNN based on the sagittal dataset. This model classified the validation set with an average accuracy of 99.2 ± 1.5%, sensitivity of 96.7 ± 6.7% and specificity of 100.0%.

**Table 1:** Subject demographics

Train and validation data: SCIENCe	SCD – Aβ negative (*n* = 101)	SCD – Aβ positive (*n* = 32)
Age	63.3 ± 7.3	68.0 ± 7.7
Male/females (*n*)	61/40	17/15
MMSE	28.9 ± 1.2	28.6 ± 1.2
Injected dose (MBq)	312 ± 37	312 ± 37
Test data: ADNI	SCD – Aβ negative (*n* = 13)	SCD – Aβ positive (*n* = 9)
Age	70.8 ± 5.1	72.7 ± 4.7
Male/females (*n*)	8/5	1/8
MMSE	29.1 ± 0.8	29.3 ± 0.7

**Table 2:** Performance metrics of the various CNNs

Train data: SCIENCe	Accuracy (%)	Sensitivity (%)	Specificity (%)
Validation data: SCIENCe			
Axial CNN	97 ± 2%	87 ± 7%	100%
Coronal CNN	95 ± 2%	83 ± 11%	99 ± 2%
Sagittal CNN	99 ± 2%	97 ± 7%	100%
Combined CNNs	97 ± 2%	87 ± 7%	100%
Test data: ADNI			
Sagittal CNN	95%	100%	92.3%

Between the two qualified readers, no differences in visual assessment of the ADNI test data exist. For this dataset the sagittal model classified with an accuracy of 95.0%, sensitivity of 100.0% and specificity of 92.3%. In addition, for this dataset an average confidence score of 4.6 ± 0.6 was given by the two qualified ^18^F-florbetapir readers and the sagittal CNN scored the scans with an average probability of 0.95 ± 0.04. The misclassified scan was scored by the qualified readers with a confidence score of 3.5 (average of the two readers) and the CNN scored the scan with a 0.88 probability.

**Fig. 4:**
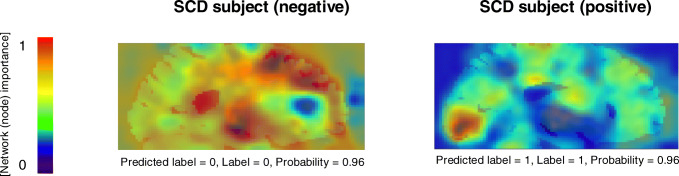
Network activation maps. For each subject, a 2D class activation map with complementary probability can be obtained. The red areas indicate patterns that are highly associated with the specific predicted class

Figure 4 shows the network attention area maps of an amyloid positive (label: 1) and amyloid negative (label: 0) SCD patient and their predicted classification. It can be seen that the occipital cortex showed high (red areas) and the frontal cortex moderate to high (yellow-orange areas) network (node) importance for the amyloid positive scan. In case of the amyloid negative scan, it can be seen that the frontal cortex showed most network (red areas) importance.

## Discussion

A 2D-CNN to discriminate between Aß negative and positive ^18^F-florbetapir PET scans of SCD subjects was successfully trained, validated and externally tested. The sagittal 2D-CNN was able to discriminate between Aß negative and positive ^18^F-florbetapir PET scans with high performance in cognitively normal SCD subjects, in which Aß deposition can be subtle or near borderline [31]. As such, the sagittal 2D-CNN can be used as a classifier of Aβ positive and negative ^18^F-florbetapir scans in SCD patients and can support the visual assessment of these scans.

In this study, we preferred the use of 2D CNNs which are able to address the increasing complexity and quantities of imaging data, while minimizing computational cost. The predictions from the different decompositions can be combined to obtain a final combined classification. However, based upon our findings, performance of the combined classification was highly dependent on the individual performance of the CNNs. Since the axial and coronal CNNs scored lower than the sagittal CNN, a combined method did not benefit from the different decompositions and we finally proposed a 2D-CNN using sagittal slices as input. Previous studies proposed 3D CNNs to predict whether the PET and/or MRI scans were from a healthy control, MCI or AD patient [7, 32, 33]. However, the ability to obtain interslice context (3D) comes at high computational cost due to the increased number of parameters used by the CNN layers. Another consideration is whether the performance of classification would benefit from these interslice features. As can be seen from the results, the sagittal 2D-CNN already performed with very high accuracy. Consequently, the use of a 3D-CNN for this specific classification task can be speculative.

In this study we used several strategies to avoid overfitting, because overfitting is a critical challenge in training deeper CNN models with a relatively limited amount of training data compared with the large number of learnable features. To resolve this, dropouts are used after the max-pooling layers. The number of convolution layers and max-pooling layers that are used in the CNN also has influence on possible overfitting. Low spatial resolution in PET enables the use of fewer CNN layers, such that the CNN has less learnable parameters and thus is less sensitive to overfitting. Therefore, the model was restricted to four convolution layers.

Other artificial intelligence-based methods have been used for the classification of amyloid PET studies, such as methods based on feature extraction in combination with machine learning [7, 8]. Machine learning based on VOI feature extraction however, ignores some small abnormal changes and these small changes can contain importantinformation that may reduce model robustness. In addition, potentially relevant brain regions might not fit into the pre-defined VOIs, limiting the representativeness of extracted features. The proposed deep learning framework uses convolution layers instead, which can jointly learn and discriminate the image features for supervised (using only the ground truth label) image classification, and could therefore have a better representation of the actual data than the predefined features. Even though our pipeline is dependent on the pre-processing, the pre-processing was done to improve generalization of multicentre scans and remove voxels outside the brain, thereby improving CNN performance.

### Model output verification/interpretation

The robustness of machine and deep learning models highly depends on the validity of the provided ground truth. Visual assessments (as ground truth) can contain errors, especially when it requires high expertise and experience. In this study, visual reads of the ^18^F-florbetapir PET scans were done by an expert nuclear medicine physician (BB) with over 15 years of experience. Ideally, visual reads should be done by more than one reader. However, the ADNI database does not provide such reads; therefore, a second qualified reader (SV) visually read and labelled the external test PET scans from the ADNI database. Between the two qualified readers, no differences in visual assessment of the external test data were observed and we therefore did not involve more readers. From the external ADNI test result, it can be seen that there was only one misclassification. However, this misclassification was out of all the external test scans scored with the lowest confidence by the readers and with a relatively low probability by the sagittal CNN. Thus, this result may suggest that the CNN assesses the scans in an almost similar fashion as the two readers. However, this should be assessed in a more extensive study, and therefore, should be interpreted with caution. Yet, a well-trained CNN might be superior to readers who lack training and experience in visually reading ^18^F-florbetapir PET studies and it is less time-consuming.

Defining AD as a biological construct provides a precise approach to target disease process and one of the first pathological changes that occurs in the brain is the accumulation of Aβ [34]. Thus, an accurate characterization and understanding of the abnormalities for Aβ deposition is important to identify early disease stages. To aid this, network maps indicating neuronal weights can be generated by combining the convolution layers and the global average-pooling layer. The network attention area maps are, however, not necessarily a measure for Aβ deposition but a representation of the extracted patterns that are important to the predicted group. The occipital cortex is a region with high non-specific binding and not commonly inspected during ^18^F-florbetapir clinical PET reading [35]. Yet, interestingly we found that this region showed high importance for the predicted group. This, however, might not be the result of increased ^18^F-florbetapir uptake, but could also be due to specific texture or shape which is associated with the predicted group. The frontal cortex is a region associated with early Aβ deposition, and therefore, used for ^18^F-florbetapir PET diagnoses [35]. From the network activation map we found that this region showed moderate to high importance for both the positive and negative amyloid scan. This could be the result of respectively increased or decreased ^18^F-florbetapir uptake in this region.

Besides ^18^F-florbetapir, ^18^F-florbetaben, ^18^F-flutemetamol and Pittsburgh Compound-B (^11^C-PiB) are other ligands to detect Aβ burden [36, 37]. In future studies, it is therefore of interest to evaluate whether the ^18^F-florbetapir-derived sagittal CNN could be used to classify Aβ scans obtained with ^18^F-florbetaben, ^18^F-flutametamol and ^11^C-PiB.

## Conclusion

A sagittal 2D-CNN to classify Aß negative and positive ^18^F-florbetapir PET scans in SCD patients was successfully constructed, trained, validated and tested. This CNN might therefore be useful for classification of Aß negative and positive ^18^F-florbetapir PET scans in situations where there is lack of trained and experienced readers.

## Data Availability

The datasets used and/or analysed during the current study are available from the corresponding author on reasonable request.
